# Incidence of pleural effusion with dasatinib and the effect of switching therapy to a different TKI in patients with chronic phase CML

**DOI:** 10.1007/s00277-024-05760-6

**Published:** 2024-04-18

**Authors:** Akriti G Jain, Quinto Gesiotto, Somedeb Ball, Lisa Nodzon, Amanda Rodriguez, Onyee Chan, Eric Padron, Andrew Kuykendall, Rami Komrokji, David A. Sallman, Jeffrey E Lancet, Javier Pinilla-Ibarz, Kendra Sweet

**Affiliations:** 1 Taussig Cancer Institute, Cleveland Clinic, 9500 Euclid Avenue CA-60,, Cleveland, OH 44195 USA; 2 Hematology Oncology Fellow, Thomas Jefferson University, Philadelphia, PA USA; 3 Vanderbilt University Medical Center, Nashville, TN USA; 4 Division of Malignant Hematology, Moffitt Cancer Center and Research Institute, Tampa, FL USA

**Keywords:** Chronic myeloid leukemia, Tyrosine kinase inhibitors, TKI, CML, Adverse events, Dasatinib

## Abstract

Dasatinib is one of the second generation tyrosine kinase inhibitors (TKI) which is approved for the treatment of patients with chronic phase CML (CP-CML) both in the front line and in the second line setting. Pleural effusion (PE) is a unique toxicity associated with dasatinib use. Our aim was to study the incidence of pleural effusion in our cohort of patients who were treated with dasatinib for CP-CML and the safety upon TKI switch. A total of 390 patients were treated with dasatinib during their course of treatment for CP-CML. A total of 69 patients (17.6%) developed any grade of PE. About 33 (48%) patients developed CTCAE grade 2 PE, 34 (49%) grade 3 and only 1 patient developed grade 4 PE. Recurrence of PE was observed in 34 (49%) patients. While only 12 patients (17.3%) continued using dasatinib after development of PE, dasatinib was discontinued in the other 57 patients. Therapy was switched to bosutinib in 13 patients out of which 6 (46%) patients re-developed PE. While only 12.5% patients developed re-accumulation of pleural fluid in patients switched to imatinib, none of the patients switched to nilotinib re-developed PE. A change in TKI to bosutinib was associated with a 46% risk of recurrence of PE in patients who develop PE on dasatinib for the treatment of CP-CML. The incidence of recurrent PE was markedly lower in patient switched to imatinib or nilotinib.

## Introduction

Chronic myeloid leukemia (CML) is characterized by the Philadelphia chromosome, a reciprocal translocation between chromosomes 9 and 22 [1]. This results in the fusion gene *BCR::ABL1* producing a chimeric protein with constitutive activity of the ABL tyrosine kinase domain leading to the development of CML. The treatment for CML was revolutionized by the development of BCR-ABL tyrosine kinase inhibitors (TKIs) which led to a markedly superior outcome compared to prior therapeutic options [2]. Although treatment free remission (TFR) has emerged as a goal in the management of CML in the recent years, the majority of patients ultimately require lifelong TKI therapy. Hence, identification of known and previously unrecognized adverse events remains essential.

Dasatinib is a second generation, oral ABL kinase inhibitor that can bind to both the active and inactive conformations of the ABL kinase domain [3, 4]. It is also active against PDGFRβ, KIT and SRC family kinases. It is approved for the treatment for newly diagnosed and resistant/intolerant CML [5]. Dasatinib is a highly potent TKI with a distinctive safety profile. Pleural effusion (PE) is a well-known adverse event associated with dasatinib use. The exact mechanisms leading to the accumulation of PE are not known, however the fluid is frequently exudative with the accumulation of lymphocytes and chyle in the exudate which led to the proposition that immune mediated mechanisms may play a role in the development of PE [6–8]. Other proposed mechanisms of PE include the off-target effects of dasatinib involving inhibition of the other kinases, especially SRC and PDGFRβ which lead to changes in vascular endothelial permeability and interstitial fluid pressure [9, 10]. The annual risk of development of PE with dasatinib was reported to be 6–9% in DASISION and 5–15% in CA180-034. At the 5- and 7-year follow up it was shown that 28% and 33% patients developed PE in DASISION and CA180-034, respectively [5, 8, 11]. Even so, PE led to discontinuation of the drug in only 6% and 7% of patients in DASISION and CA180-034 [5, 11].

Bosutinib is another second generation dual SRC/ABL TKI. Clinical studies with bosutinib have reported frequent gastrointestinal and cutaneous adverse events, and some of the toxicities with bosutinib suggest a unique adverse event profile with the drug compared to other TKIs [12]. Bosutinib has minimal inhibition of PDGFβ [13]. Thus, for patients who develop PE while on treatment with dasatinib if change in therapy is warranted, bosutinib is a common selection. Recently, a switch to bosutinib after development of PE while on dasatinib has been shown to be associated with a 30% risk of developing recurrent PE [14]. The aim of our study was to identify the incidence of pleural effusions in a cohort of patients treated with dasatinib for chronic phase (CP)-CML, and the safety of switching therapy to bosutinib compared to other TKIs.

## Methods

This is a retrospective study including all patients treated with dasatinib for CP-CML at Moffitt Cancer Center between 1992 and 2021. Data including baseline patient characteristics, date of diagnosis, line of treatment with details including date of initiation and termination of treatment, reason for discontinuation and dose were collected. The study was approved by the Moffitt Cancer Center institutional review board. Patients were treated in line with United States Food and Drug Administration indications and the drug label. Informed consent from each individual was not obtained because this is a retrospective chart review. Each individual provided consent for treatment and details on treatment and side effects was obtained using patient charts.

PE was graded according to the common terminology criteria for adverse events (CTCAE) v5.0. A descriptive analysis of the data collected is reported. Chi-square test was used for categorical variables.

## Results

A total of 390 patients, 184 males and 206 females, were treated with dasatinib during their course of treatment for CP-CML. The median age at diagnosis of CML was 50 years. Table 1 describes the baseline characteristics of the patients included in this study. 79% of patients were Caucasian. Dasatinib was used as front-line therapy in 150 (38.4%) patients, second line in 177 (45.3%) patients and third line or above in 63 (16.2%) patients.

**Table 1:** Baseline patient characteristics

Characteristic		Total (*n* = 390)
Median age at Diagnosis, years (range)		50 (12–84)
Gender		
	Male	184 (47.2%)
	Female	206 (52.8%)
Dasatinib use		
	Front Line	150 (38.4%)
	Second Line	177 (45.4%)
	Third Line or beyond	63 (16.2%)

**Fig. 1:**
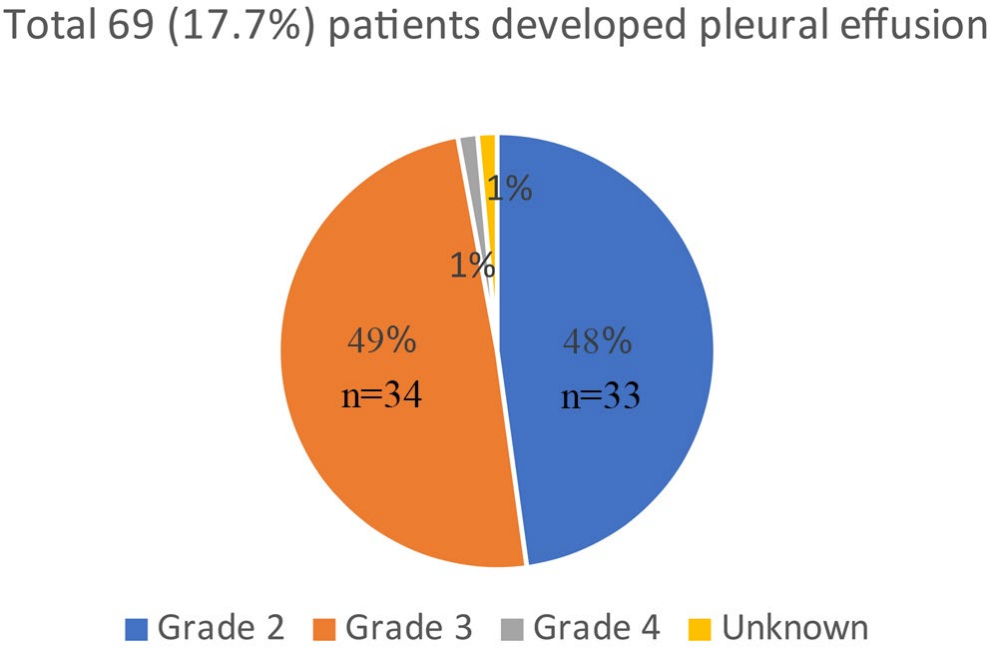
Number of patients that developed pleural effusion while on dasatinib

A total of 69 patients (17.6%) developed any grade of PE (Fig. 1). Thirty-three (48%) patients developed CTCAE grade 2 PE, 34 (49%) grade 3, only 1 patient developed grade 4 PE and the grade of PE for 1 patient is not known. Recurrence of PE was observed in 34 (49%) patients. The dose of dasatinib for the patients who developed PE was 140 mg (6%), 100 mg (71%), 70 mg BID (4.3%), 70 mg (3%), 20 mg (3%) and unknown (13%). Therapy was changed to another TKI without first trying a dasatinib dose modification in 43% patients. Alternatively, the dasatinib dose was initially reduced in 46% patients. Data is not available for the remaining 11% of patients. The incidence of PE was significantly higher in patients ≥ 65 years old compared to patients < 65 years old (31.3% vs. 13.1%; *p* < 0.001). Non-smokers were more likely to develop PE compared to current or former smokers (26.4 vs. 10 or 13.6%; *p* = 0.006). Gender, race, BMI or line of dasatinib use did not affect the rate of development of PE (Table 2). PE directed treatment included a combination of furosemide (49%), steroids (20%) and thoracentesis (49%). The average time from initiation of dasatinib to development of PE was 31 months (median 15 months, range 0-161 months).

**Table 2:** Patient characteristics and the risk of developing pleural effusion (PE)

Variable		Total *n* = 390 (%)	Developed PE *n* = 69 (%)	No PE *n* = 321 (%)	p-value
Age at initiation of Dasatinib					< 0.001
	≥ 65 years	99 (25.4)	31 (31.3)	68 (68.7)	
	< 65 years	291 (74.6)	38 (13.1)	253 (86.9)	
Gender					0.344
	Male	184 (47.2)	29 (15.8)	155 (84.2)	
	Female	206 (52.8)	40 (19.4)	166 (80.6)	
Race					0.221
	White	308 (78.9)	61 (19.8)	247 (80.2)	
	African American	33 (8.4)	5 (15.1)	28 (84.9)	
	Asian	3 (0.7)	0	3 (100)	
	Hispanic	12 (3.3)	1 (8.3)	11 (91.7)	
	Unknown/Others	34 (8.7)	2 (5.9)	32 (94.1)	
Dasatinib – Line of Therapy					0.924
	Front line	150 (38.4)	27 (18)	123 (82)	
	Second line	177 (45.4)	30 (16.9)	147 (83.1)	
	Third line or beyond	63 (16.2)	12 (19)	51 (81)	
Tobacco Use					0.006
	Current Smoker	30 (7.7)	3 (10)	27 (90)	
	Former Smoker	213 (54.6)	29 (13.6)	184 (86.4)	
	Never Smoker	121 (31)	32 (26.4)	89 (73.6)	
BMI (kg/m^2^)					0.413
	Underweight (< 18.5)	3 (0.8)	1 (33.3)	2 (66.7)	
	Normal (18.5 – <25)	98 (25.1)	16 (16.3)	82 (83.7)	
	Overweight (25 – <30)	134 (34.3)	29 (21.6)	105 (78.4)	
	Obese (≥ 30)	154 (39.5)	23 (14.9)	131 (85.1)	

Looking at CML therapy post development of PE, only 12 patients (17.4%) ultimately continued using dasatinib long-term after development of PE, and dasatinib was eventually discontinued in the other 57 patients (Table 3). In the 12 patients that continued using dasatinib the dose was reduced to 50 mg/day. Median duration of therapy on dasatinib was 39 months in patients who developed PE. Treatment was switched from dasatinib to bosutinib in 13 patients. Of these, 6 (46%) patients re-developed PE. The median duration on therapy for patients switched to bosutinib that did not develop recurrent PE was 46 months and 12.5 months for patients that experienced recurrence of PE after switching to bosutinib. The median time to re-accumulation of pleural effusion after switching to bosutinib was 9 months. In looking at patients who switched from dasatinib to other TKIs, only 12.5% of patients placed on imatinib (*n* = 1/8) developed re-accumulation of pleural fluid, and none of the patients who were switched to nilotinib (*n* = 23) re-developed PE (*p* = 0.0027).

**Table 3:** CML therapy post pleural effusion and rates of re-accumulation of pleural effusion

CML Therapy post pleural effusion		Total *n* = 69	Re-accumulation of PE, n (%)
Continued Dasatinib		12	
Switched Therapy to another TKI			
	To Bosutinib	13	6 (46.1)
	To Imatinib	8	1 (12.5)
	To Nilotinib	23	0
	To Ponatinib	2	0
Others		11	

*Others include patients that stopped TKI as they met TKI discontinuation criteria and patients that were enrolled on clinical trials

## Discussion

To the best of our knowledge, this is the largest retrospective study to demonstrate an increased incidence of PE after switching to bosutinib in CP-CML patients who have experienced PE on dasatinib outside of clinical trials. The first retrospective analysis by Tiribelli, et al. included 20 patients who experienced PE on dasatinib and were then switched to bosutinib. They reported a 30% (6/20) rate of PE after the switch [14]. Our data identified 46.1% (6/13) of patients with recurrent PE with bosutinib and this was significantly higher compared to other TKIs (*p* = 0.0027).

The overall rate of PE with dasatinib in this study was lower than what was reported in DASISION (17.7% vs. 28%). Further differences lie in the CTCAE v5.0 grading of PE between the two reports. As an example, 8.4% (*n* = 33) of patients developed grade 2 PE in our study while 26% (*n* = 66) experienced grade 1 or 2 PE in DASISION. We observed a modest increase in the rate of grade 3 or 4 PE compared to DASISION (9% vs. 3%, respectively). Age, twice daily dosing, history of cardiac disease, hypertension, hypercholesterolemia, autoimmune disease, and skin rash during prior treatment with imatinib or dasatinib are among the previously reported risk factors for PE generation under dasatinib therapy [9, 15–17]. Hence, it is recommended to avoid dasatinib in patients with respiratory failure, and previous or concomitant pleural or pericardial disease. Similar to DASISION the percentage of patients who developed PE was higher in patients age ≥ 65 years compared to their younger counterparts (31.3% vs. 13.1%; *p* < 0.001). However, our rates were not as disparate as DASISION (60% ≥65 years vs. 25% <65 years). Interestingly, we also found that more non-smokers developed PE compared to current or former smokers, which has not been reported in the past. Of the total 390 dasatinib treated patients, 57 (14.6%) discontinued the drug as a result of pleural effusion, which is higher than the number seen in DASISION (6%, *n* = 15). Recent guidelines on management of PE can help in streamlining treatment of PE and if effectively managed, some patients may continue on dasatinib [9]. In addition, recent studies have shown that lower dose (50 mg/day) of dasatinib can lead to similar or even better response rates with much lower rates of adverse events [18, 19]. In the propensity matched analysis by Jabbour et al., authors from MD Anderson showed that the rate of pleural effusion with 50 mg/day of dasatinib was only 5% compared to 21% in patients treated with 100 mg of Dasatinib for frontline treatment of CP-CML [19]. Hence, dose reduction might be a another way of managing adverse events rather than switching to another TKI which might also be at full dose which can lead to recurrence of the adverse event.

The phase 4 BYOND study reported that 16.6% patients (*n* = 27) experienced PE on bosutinib with a 6.1% grade 3 or 4 PE [20]. It is important to note, however, that the patient population enrolled in this phase 4 study were heavily pretreated with 60.9% of patients having had prior exposure to dasatinib. This may have been a contributing factor to the high rate of PE seen in BYOND [20]. At 5 years, the incidence of PE with bosutinib was reported to be 6% in the BFORE trial [21]. There have been recent reports on cross-intolerance with TKIs. Rates of recurrent PE on bosutinib after PE with dasatinib have been reported between 28 and 52% [22–25]. Our rate of 46% falls within that range. In a Spanish study on 62 CP-CML patients receiving bosutinib as fourth line treatment, recurrent PE with bosutinib after experiencing PE with dasatinib was shown to be 28% (7/25) and this led to discontinuation of bosutinib in only 2 patients [22]. In the phase I/II trial of bosutinib, 12 out of the 23 patients (52%) that previously suffered PE with dasatinib experienced recurrent PE leading to discontinuation of bosutinib in 2 patients [24]. Another French report noted worsening of PE in 2 out of 4 patients after switch from dasatinib to bosutinib [23]. Some other case reports have also reported recurrent PE after switch from dasatinib to bosutinib after experiencing PE with dasatinib [25].

The median time to recurrence of PE on bosutinib after switching from dasatinib in our study was 9 months which is longer compared to 3 months reported by Tiribelli, et al. [14]. However, in both studies, this time to re-accumulation while on bosutinib was shorter compared to the time to PE on dasatinib prior to the switch; 31 months in our study and 20 months in the study by Tiribelli, et al. [14].

Limitations of our study include retrospective design, small number of patients who were switched from dasatinib to bosutinib and lack of efficacy data. Despite the above limitations we did show an increased incidence of recurrent PE in a cohort of patients who were switched to bosutinib after developing PE with dasatinib. The increase in the rate of recurrence of PE in patients switched to bosutinib was significantly higher than the patients switched to other TKIs including imatinib or nilotinib. This is in line with previous studies and perhaps in patients with concomitant risk factors like advanced age and concomitant cardio-pulmonary disease after experiencing PE with dasatinib, bosutinib should be avoided. Further prospective studies are needed to determine the safety of bosutinib in this setting.

## Data Availability

Due to the comprehensive nature of our CML database, we will not be able to share data.
